# Monocyte distribution width enhances early sepsis detection in the emergency department beyond SIRS and qSOFA

**DOI:** 10.1186/s40560-020-00446-3

**Published:** 2020-05-05

**Authors:** Elliott D. Crouser, Joseph E. Parrillo, Greg S. Martin, David T. Huang, Pierre Hausfater, Ilya Grigorov, Diana Careaga, Tiffany Osborn, Mohamad Hasan, Liliana Tejidor

**Affiliations:** 1 Division of Pulmonary and Critical Care Medicine, The Ohio State University Wexner Medical Center, 201 Davis Heart & Lung Research Institute, 473 West 12th Avenue, Columbus, OH USA; 2 Heart and Vascular Hospital, Hackensack University Medical Center, Hackensack, NJ USA; 3 Division of Pulmonary, Allergy, Critical Care and Sleep Medicine, Emory University and Grady Memorial Hospital, Atlanta, GA USA; 4 Department of Critical Care Medicine, School of Medicine, University of Pittsburgh, Pittsburgh, PA USA; 5 Emergency Department, GRC-14 BIOSFAST and UMR 1166 IHU ICAN, APHP-Sorbonne Université Hospital, Pitié-Salpêtrière site, Sorbonne Université, Paris, France; 6 Beckman Coulter, Inc., Brea, CA USA; 7 Beckman Coulter, Inc., Miami, FL USA; 8 Division of Emergency Medicine, Barnes Jewish Hospital, Washington University School of Medicine, Saint Louis, MO USA

**Keywords:** Biomarker, Blood, Sepsis-2, Sepsis-3, Severe sepsis, Infection, ED

## Abstract

**Background:**

The initial presentation of sepsis in the emergency department (ED) is difficult to distinguish from other acute illnesses based upon similar clinical presentations. A new blood parameter, a measurement of increased monocyte volume distribution width (MDW), may be used in combination with other clinical parameters to improve early sepsis detection. We sought to determine if MDW, when combined with other available clinical parameters at the time of ED presentation, improves the early detection of sepsis.

**Methods:**

A retrospective analysis of prospectively collected clinical data available during the initial ED encounter of 2158 adult patients who were enrolled from emergency departments of three major academic centers, of which 385 fulfilled Sepsis-2 criteria, and 243 fulfilled Sepsis-3 criteria within 12 h of admission. Sepsis probabilities were determined based on MDW values, alone or in combination with components of systemic inflammatory response syndrome (SIRS) or quick sepsis-related organ failure assessment (qSOFA) score obtained during the initial patient presentation (i.e., within 2 h of ED admission).

**Results:**

Abnormal MDW (> 20.0) consistently increased sepsis probability, and normal MDW consistently reduced sepsis probability when used in combination with SIRS criteria (tachycardia, tachypnea, abnormal white blood count, or body temperature) or qSOFA criteria (tachypnea, altered mental status, but not hypotension). Overall, and regardless of other SIRS or qSOFA variables, MDW > 20.0 (vs. MDW ≤ 20.0) at the time of the initial ED encounter was associated with an approximately 6-fold increase in the odds of Sepsis-2, and an approximately 4-fold increase in the odds of Sepsis-3.

**Conclusions:**

MDW improves the early detection of sepsis during the initial ED encounter and is complementary to SIRS and qSOFA parameters that are currently used for this purpose. This study supports the incorporation of MDW with other readily available clinical parameters during the initial ED encounter for the early detection of sepsis.

**Trial registration:**

ClinicalTrials.gov, NCT03145428. First posted May 9, 2017. The first subjects were enrolled June 19, 2017, and the study completion date was January 26, 2018.

## Introduction

Sepsis is a leading cause of mortality and healthcare expenses worldwide. Healthcare organizations such as the World Health Organization and Centers for Disease Control and Prevention have designated sepsis as a high-priority disease, including measures to improve the prevention, diagnosis, and management of sepsis [1, 2]. Current guidelines emphasize the benefits of early detection and treatment, as each hour of antibiotic treatment delay during early severe sepsis is associated with 4–9% mortality increase [3–5]. The vast majority of sepsis cases (> 85%) are present at the time of admission to the hospital [6], and clinical deterioration from non-severe sepsis to life-threatening sepsis can occur rapidly [7–10]. Unfortunately, it is difficult to distinguish sepsis from non-infectious causes of acute illness in the ED setting because these patients often share common clinical signs and symptoms [11]. Moreover, even in hospitals with early sepsis detection notification processes, also referred to as “code sepsis” alerts, sepsis detection is often delayed in the ED leading to suboptimal treatment, longer ICU length of stay, and higher mortality [12, 13].

Early sepsis detection in the ED relies upon clinical data that is readily available during the initial patient presentation [14]. Four clinical parameters meet these criteria and are commonly used to identify sepsis during the initial patient encounter: abnormal white cells blood count (WBC), tachycardia, tachypnea, and fever (or hypothermia), collectively designated as systemic inflammatory response syndrome (SIRS) criteria [15]. WBC is a component of complete blood count (CBC), blood tests that are often obtained during the initial ED encounter that often influences decisions to admit the patient to the hospital [16]. Alternatively, three easily detected clinical parameters, tachypnea, altered mental status, and hypotension, can be combined into quick organ failure assessment (qSOFA) score to identify early signs of organ failure, associated with severe sepsis [17–20].

Monocyte distribution width (MDW) is a hematologic parameter describing the changes in the size distribution of circulating monocytes which can be reported as a part of the routine CBC with differential on certain Beckman Coulter DxH series hematology analyzers. We previously reported that MDW determined during the initial ED encounter predicted higher probability of sepsis within 12 h of ED admission with ROC AUC of 0.79 (95% CI = − 0.76–0.82) using Sepsis-2 and 0.73 (95% CI = 0.69–0.76) using Sepsis-3 criteria [21, 22]. In many cases, the septic patients with abnormal MDW did not meet criteria for sepsis at the time of initial ED evaluation, suggesting that MDW is a disease-specific marker that is predictive of progression from localized infection to sepsis. The previously published analysis was limited to assessing the value of MDW and WBC parameters [21], irrespective of the patient’s SIRS or qSOFA score at presentation, without addressing how MDW could be interpreted as part of the comprehensive early ED assessment.

The objective of this analysis was thus to determine the value that MDW contributes to the diagnostic accuracy of parameters comprising SIRS and qSOFA scores assessed at the time of the initial ED presentation. We hypothesized that adding MDW to standard clinical assessment and risk-scoring modulates the probability of sepsis predicted from clinical parameters alone and thus can be used to improve early detection of sepsis in the ED.

## Methods

### Clinical trial protocol

To determine the predictive value of MDW for sepsis when combined with the presenting clinical parameters that are commonly used to screen for the initial detection of sepsis in the ED (SIRS or qSOFA), we performed a retrospective analysis of a clinical trial sponsored by Beckman Coulter, Inc. [ClinicalTrials.gov (NCT03145428) and approved by the Western Institutional Review Board, Inc. (protocol # C03747)]. The trial was conducted between June 19, 2017, and January 26, 2018, at three academic centers: Hackensack University Medical Center (Hackensack, NJ), The Ohio State University Wexner Medical Center (Columbus, OH), and University of Pittsburgh Medical Center Shadyside Hospital (Pittsburgh, PA) and led to US Food and Drug Administration (FDA) clearance and the European Union’s In Vitro Diagnostic Directive (CE-IVDD) CE Mark certification of MDW for early detection of sepsis in adult ED patients.

**Fig. 1:**
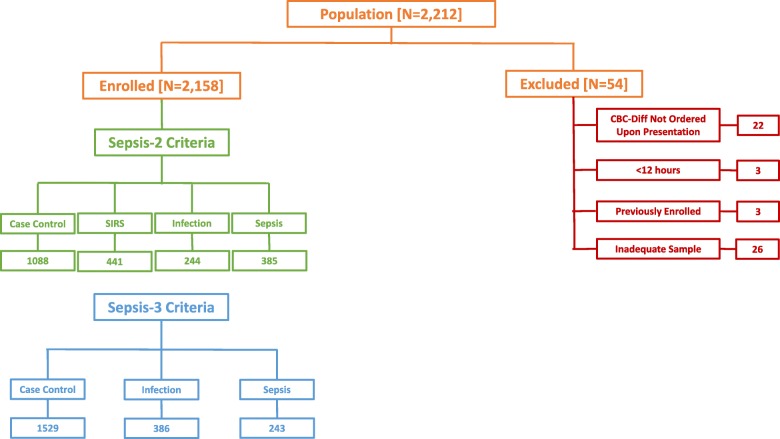
Flow diagram describing patient screening and enrollment. The study was conducted between April 2017 and January 2018. 2.5% of subjects screened were excluded for various reasons, as noted above, such that 97.5% of subjects screened were enrolled in the study

Informed consent was waived for this study based on the de-identification of all data using honest brokers. The study enrolled 2158 adults in the ED, age 18 to 89 years, whose evaluation included a CBC with differential upon presentation to the ED. Exclusion criteria were inadequate blood samples (e.g., analyzed > 2 h after collection), discharged from the ED within 12 h (i.e., incomplete data for sepsis classification), prisoners, and prior study enrollment (Fig. 1).

All blood samples were analyzed on a UniCel® DxH 800 hematology analyzer (Beckman Coulter, Inc.) within 2 h of collection. This instrument has a unique capability to measure specific cell volume parameters and the distribution of cell volumes within a group of cells, as previously reported [22, 23].

### Sepsis definitions

Study subjects were categorized based upon the “Sepsis-2” consensus criteria [24]: non-SIRS (i.e., zero or one *SIRS criterion) and no infection, SIRS (≥ 2 SIRS and no infection criteria), Sepsis (infection plus SIRS) [inclusive of Sepsis (no organ failures), Severe Sepsis (sepsis with one or more organ failures), and Septic Shock (sepsis with refractory hypotension)], and Infection but no sepsis (i.e., zero or one SIRS criterion), and by Sepsis-3 criteria [25]: controls, infection, and sepsis (based upon sequential organ failure assessment (^†^SOFA) criteria). The presence of infection was determined based upon retrospective chart review of tests performed and clinical data available within the first 12 h of ED presentation. If no workup for infection was initiated within 12 h, the patient was categorized as “not infected” by the adjudicator. *SIRS criteria are as follows: WBC > 12,000 or < 4000 or > 10% bands, pulse > 90, respiratory rate > 20, and temperature < 96.8 °F or > 100.4 °F; ^†^SOFA or sequential organ failure assessment score [26].

The prevalence of patients meeting Sepsis-2 criteria in the study population was 17.8%, exceeding sepsis prevalence reported in non-differentiated ED population [14, 27, 28]. This can be explained by the enrollment criteria requiring a minimum of 12-h ED stay and CBC with differential order. Based on prior reports, approximately 39% of patients presenting to the ED had their CBC evaluated [29] and the prevalence of sepsis within that sub-population was reported to be 5–10% [22, 28, 29].

### Statistical methods

Current practice for early sepsis detection often relies on clinician’s assessment of sepsis probability in patients presenting with otherwise non-specific signs and symptoms. To reflect such an approach, this study analyzed the probability and likelihood ratios for sepsis based upon the values of MDW in combination with other SIRS parameters determined during the initial patient encounter in the ED (typically within the first 2 h of ED admission). Diagnostic ability for each clinical parameter was evaluated based on sensitivity, specificity positive predictive value (PPV), and negative predictive value (NPV) calculated at predetermined cut-offs as shown in Table 2. The predicted probability of a positive sepsis diagnosis was calculated from the positive likelihood ratios (LRs+) as described elsewhere [25, 30, 31]. In this approach, predicted sepsis probability after receiving test results, or post-test probability *P*_1_, is based on pre-test probability *P*_0_ and LR+ and is calculated as follows:

$$ {P}_1=\frac{P_0\times \mathrm{IR}+}{\left(1-{P}_0+{P}_0\times \mathrm{LR}+\right)} $$

where *P*_0_ is the sepsis prevalence. To account for differences in prevalence observed in trial compared to that in non-differentiated ED populations, the sepsis probabilities for each of the parameter combinations were calculated directly from the clinical study data and extrapolated to a range of pre-test probabilities using sepsis likelihood ratio (LR+) methodology as described above. The odds ratios (ratios of post-test probabilities) for sepsis diagnosis between parameter combinations with abnormal and normal MDW values were extrapolated to a sepsis prevalence of 8%.

## Results

### Patient demographics

A total of 2158 ED patients were recruited of which 385 fulfilled Sepsis-2 criteria and 243 fulfilled Sepsis-3 criteria within 12 h of ED admission. Additional demographic information is provided in Table 1, and the flow diagram for study enrollment for both Sepsis-2 and Sepsis-3 is provided in Fig. 1.

**Table 1:** Summary demographics by group

	Summary demographics by group			
	Control	SIRS	Infection	Sepsis
**Total subjects**	1088	441	244	385
**Subject age—mean (min–max)**	60 (18–89)	59 (18–89)	63 (21–89)	61 (18–89)
**Male gender, no. (%)**	529 (49)	202 (46)	107 (44)	195 (51)
**Race**				
White	731	318	181	260
Black or African American	247	90	40	82
American Indian or Alaska native	1	0	1	2
Native Hawaiian or other Pacific islander	2	0	0	0
Asian	28	9	5	11
Not provided (includes others)	79	24	17	30
**Pre-existing conditions, no. (%)**				
Immune-suppression/immune stimulant	129 (12)	80 (18)	36 (15)	88 (23)
Malignancy	132 (12)	87 (20)	41 (17)	77 (20)
Antibiotics	69 (6)	33 (7)	55 (23)	75 (19)
Alcoholism	58 (5)	29 (7)	5 (2)	8 (2)
Smoking	202 (19)	91 (21)	35 (14)	70 (18)

### Comparison of individual clinical markers for early sepsis detection in ED

The ideal sepsis biomarker would be readily available during the initial ED encounter and would be sensitive and specific for sepsis detection. However, the clinical presentation of sepsis is highly variable, such that different SIRS and qSOFA criteria are often met by different septic patients. Furthermore, the diagnostic performance of individual SIRS criteria observed in the trial was generally poor to moderate, with sensitivity ranging from 20 to 75%, and positive predictive values in the range of 33–77%, as shown in Table 2. Abnormal WBC, MDW, and tachycardia were most sensitive and the best available sepsis biomarkers.

**Table 2:** Prevalence of clinical parameters among 2158 ED patients as reflected by the sensitivity, specificity, positive predictive value (PPV), and negative predictive value (NPV) of each parameter for sepsis (Sepsis-2). The pre-test probability of sepsis in this ED population was 17.8%

Clinical parameters	Sepsis sensitivity	Sepsis specificity	Sepsis PPV	Sepsis NPV
Elevated MDW (> 20)	74.0%	72.0%	36.5%	92.7%
Abnormal WBC (< 4000 or > 12,000)	68.8%	81.6%	44.8%	92.3%
Tachycardia (HR > 90 bpm)	74.5%	67.7%	33.4%	92.4%
Elevated body temperature (< 96.8 °F or > 100.4 °F)	20.0%	98.7%	77.0%	85.0%
Tachypnea (RR > 20/min)	20.3%	93.0%	38.6%	84.3%
Hypotension (SBP ≤ 100 mmHg)	15.8%	97.1%	54.0%	84.2%
Altered mental status (GCS < 15)	11.7%	93.2%	27.1%	82.9%

### MDW augments performance of SIRS parameters for sepsis detection in ED

**Fig 2:**
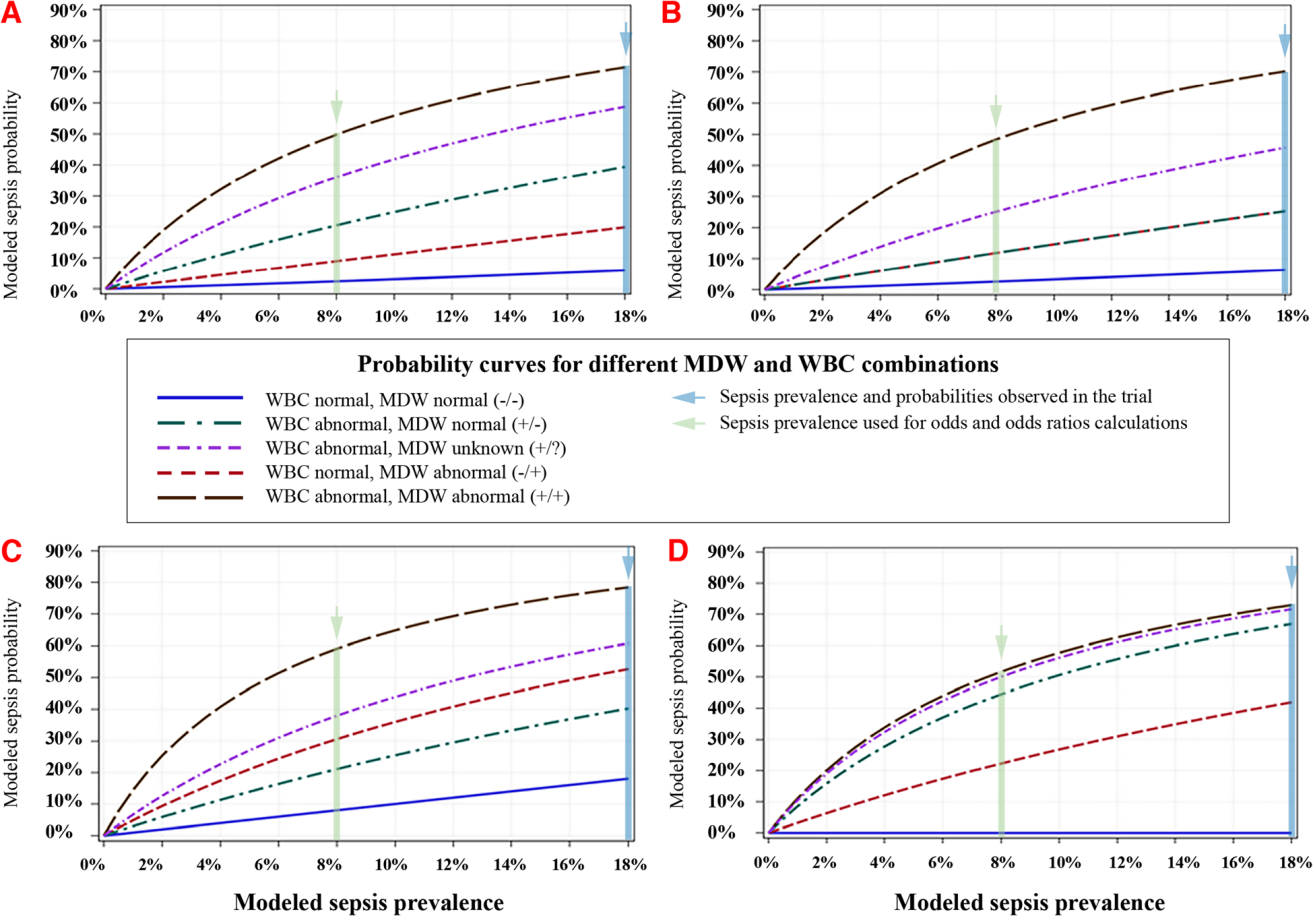
MDW improves early sepsis detection when combined with each SIRS vital sign criterion. The probability of sepsis in patients presenting initially to the ED with abnormal vital signs of tachycardia (**a**), tachypnea (**b**), both tachycardia and tachypnea (**c**), or abnormal temperature (**d**) is consistently lower if the MDW is normal (solid blue line) compared to abnormal MDW (dashed red line). The probability of sepsis is also higher when a vital sign abnormality is combined with abnormal WBC (dashed purple line). When a vital sign is abnormal along with abnormal WBC, abnormal MDW indicates higher sepsis probability (dashed black line) and normal MDW indicates lower sepsis probability (dashed green line)

MDW augments the performance when used in combination with individual SIRS components obtained during the initial ED encounter. As shown in Fig. 2, MDW (and WBC) independently enhanced the performance of the SIRS vital sign parameters of tachycardia, tachypnea, or abnormal temperature for the detection of sepsis based on post-test sepsis probability calculated for a range of sepsis prevalence to represent the variation among ED populations (Fig. 2a–d).

The relative odds of sepsis diagnosis within 12 h of ED admission were likewise influenced by initial MDW and SIRS criteria parameters, as per Table 3. Regardless of SIRS criteria, an abnormal MDW, compared to a normal MDW, increased the odds, by approximately 6-fold, of identifying patients with sepsis using Sepsis-2 criteria within 12 h of ED admission.

**Table 3:**
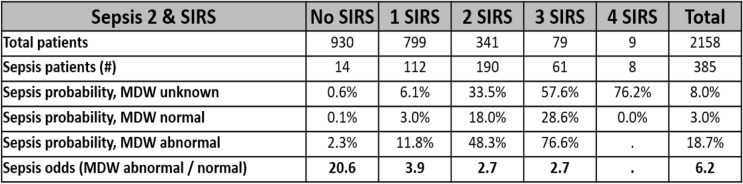
MDW improves the odds of detecting Sepsis-2 among ED patients presenting with SIRS criteria

Ninety-eight percent of the patients presenting to the ED who developed sepsis within 12 h of ED admission (Sepsis-2) had 0–3 abnormal SIRS parameters at the time of initial ED evaluation. Abnormal MDW predicted higher probability of sepsis compared to SIRS criteria alone (unknown MDW) or with normal MDW. Overall, abnormal MDW was associated with 6.2-fold higher odds of sepsis compared to normal MDW in all septic patients at the time of initial ED presentation. For this analysis, the pre-test probability for sepsis in the ED was estimated at 8%

### MDW augments performance of qSOFA components for sepsis detection in ED

**Fig. 3:**
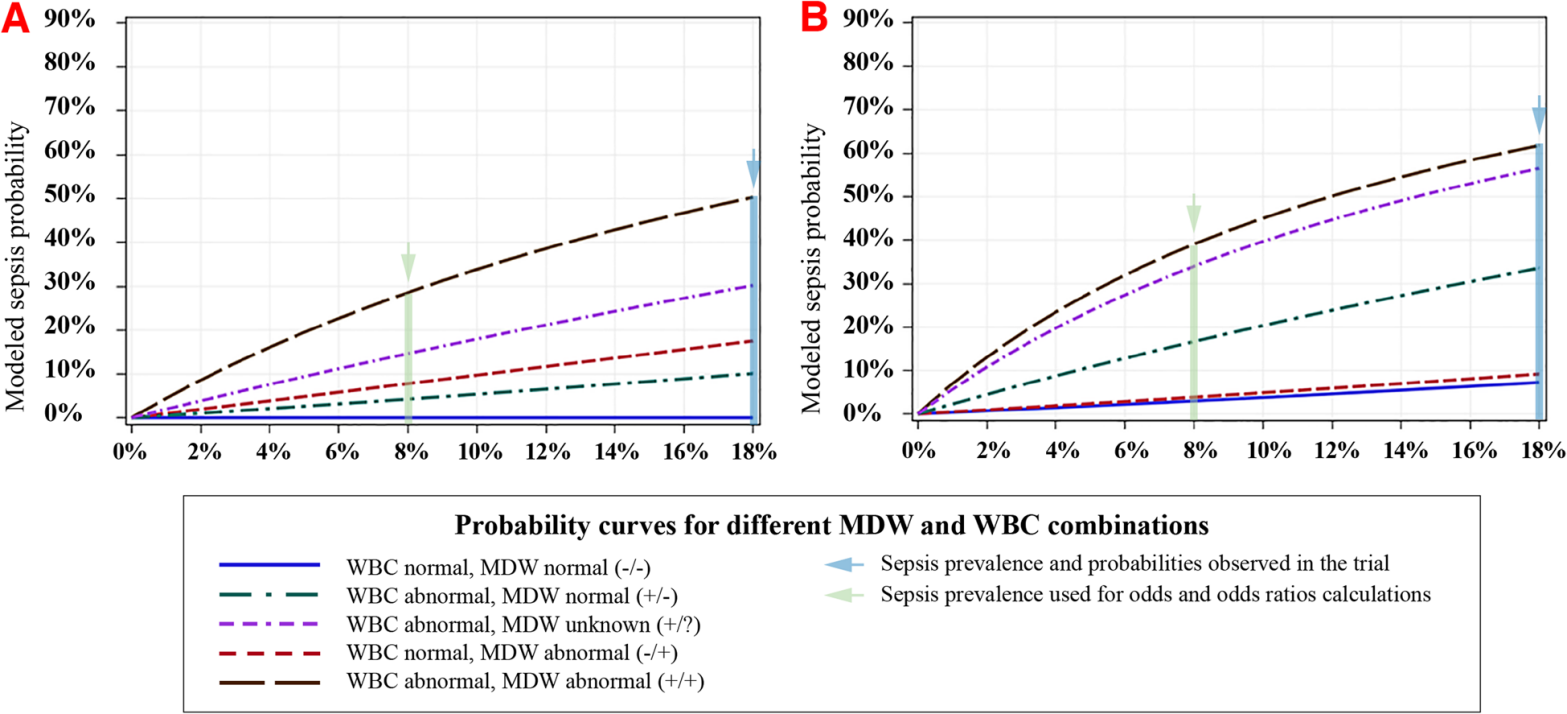
MDW improves early sepsis detection in combination with altered mental status and hypotension. The probability of sepsis in patients presenting initially to the ED with altered mental status (AMS) (**a**) is lower if the MDW is normal (solid blue line) compared to abnormal MDW (dashed red line). The probability of sepsis is also higher when AMS is combined with abnormal WBC (dashed purple line). When AMS is associated with abnormal WBC, abnormal MDW further predicts higher sepsis probability (dashed black line) and normal MDW predicts lower sepsis probability (dashed green line). In the setting of hypotension (**b**) with elevated WBC (purple dashed line), normal MDW is associated with lower sepsis risk (green dashed line)

The quick sequential organ failure assessment (qSOFA) tool is an alternative to SIRS for early sepsis detection and is based on three criteria: altered mental status, hypotension, and tachypnea. As shown in Table 2, qSOFA criteria were less common findings among septic patients equating with lower sensitivity. As shown in Fig. 2a, the probability of sepsis in patients with tachypnea was higher when MDW was abnormal vs. normal, particularly when WBC was also abnormal, thus augmenting the performance of tachypnea for sepsis detection. Abnormal MDW also improved sepsis detection among patients with altered mental status, particularly when WBC was also abnormal (Fig. 3a). Among ED patients presenting with hypotension (an uncommon presentation in the ED as per Table 1) and abnormal WBC, normal MDW predicts lower sepsis risk. Table 4 demonstrates the added value of MDW in combination with qSOFA criteria assessed during the initial ED encounter, wherein odds of Sepsis-3 criteria being met within 12 h of ED admission are strongly influenced by the initial MDW value. Overall, the odds of Sepsis-3 increased by approximately 4-fold in patients with abnormal vs. normal MDW, regardless of baseline qSOFA value.

**Table 4:**
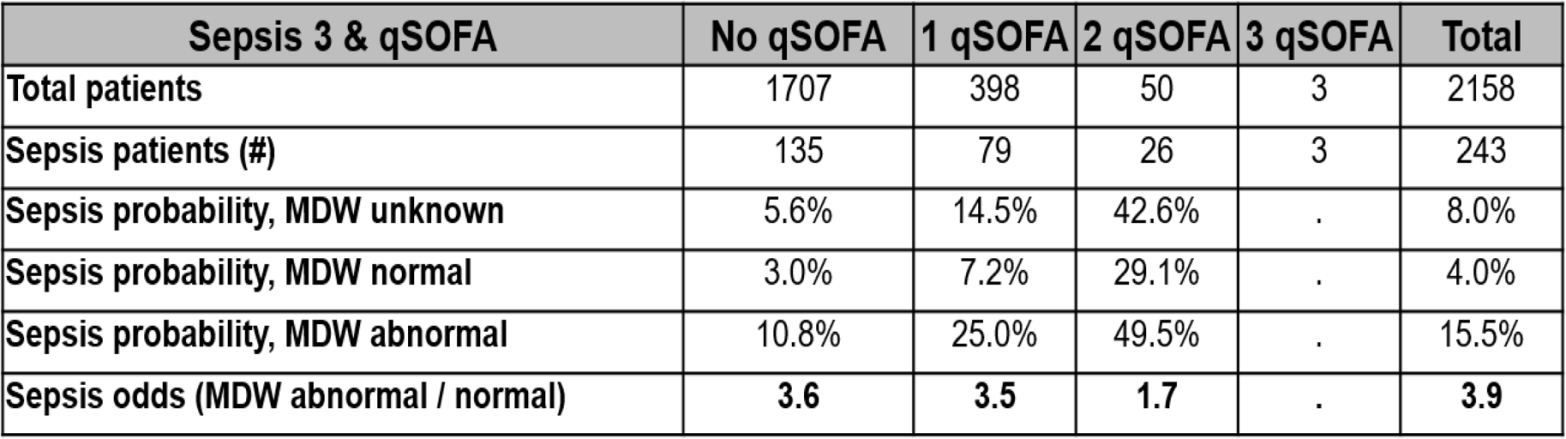
MDW improves the odds of detecting Sepsis-3 among ED patients presenting with qSOFA criteria

Most of the patients presenting to the ED who developed sepsis within 12 h of ED admission (Sepsis-2) met 0–1 qSOFA parameters at the time of initial ED evaluation. Abnormal MDW predicted higher probability of sepsis compared to qSOFA criteria alone (unknown MDW) or when MDW was normal. Overall, abnormal MDW at the time of initial ED presentation was associated with 3.9-fold higher odds of sepsis compared to normal MDW. For this analysis, the pre-test probability for sepsis in the ED was estimated at 8%

### MDW improves early sepsis detection in ED patients with normal WBC

**Fig. 4:**
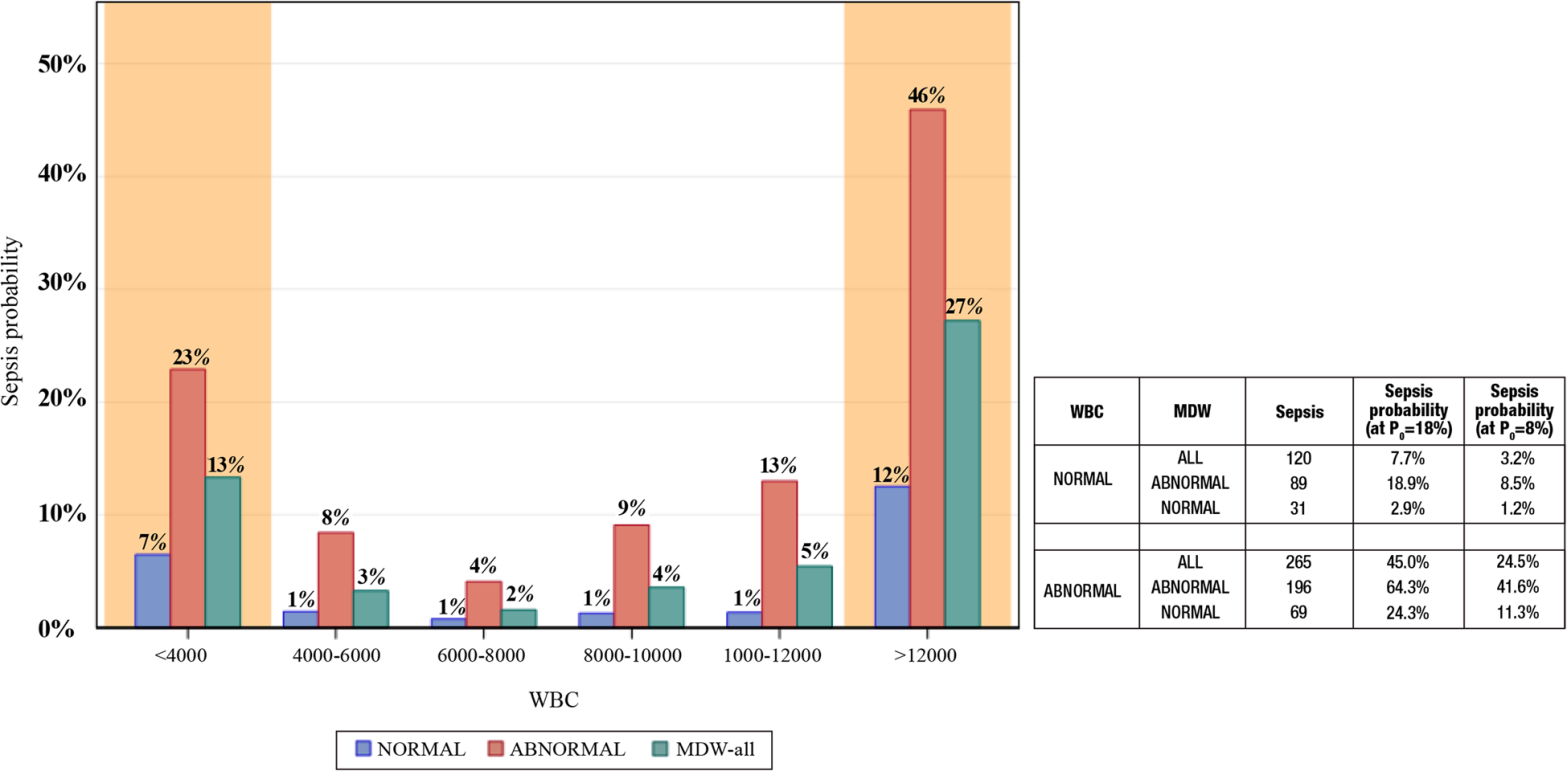
MDW improves detection of sepsis in ED patients regardless of WBC value at presentation. An elevated MDW value predicts higher sepsis probability in ED patients presenting with abnormal WBC (< 4000 or > 12,000, orange shading) and within the range of normal WBC values (4000–12,000, no shading). In contrast, a normal MDW at presentation to the ED reduces sepsis probability regardless of normal or abnormal WBC value. When all patients with normal CBC are combined, the risk of sepsis is 6-fold higher if MDW is elevated compared to those with a normal MDW value. Notably, 31% of all sepsis cases presented to the ED with a WBC in the normal range. Associated table summarizes sepsis probabilities for combinations of MDW and WBC determined in trial (*P*_0_ = 18%) and modeled at *P*_0_ = 8%. The chart numbers reflect sepsis probabilities at *P*_0_ = 8%. Abnormal WBC cohort combines patients with WBC < 4000 and WBC > 12,000

We previously reported that the combination of abnormal WBC and abnormal MDW more accurately detects sepsis compared to either abnormal parameter alone [21]. Furthermore, 31% of septic ED patients presented initially with WBC values within the normal range, and in this group, an elevated MDW predicted much higher sepsis probability and a normal MDW predicted lower sepsis probability. Regardless of the WBC level, an abnormal MDW predicted higher sepsis probability (Fig. 4).

## Discussion

MDW was previously reported to have good performance when evaluated alone or in combination with WBC for sepsis detection within 12 h of ED admission [21, 22]. We now further demonstrate in this study that MDW augments sepsis detection when used in combination with other readily available clinical parameters during the initial encounter, spanning the first hours, in a large cohort of ED patients. Compared to other clinical parameters that are predictive of sepsis in its early stages, such as fever, altered mental status, and hypotension, MDW elevation is more common among septic patients in the ED (Table 3), which contributes to improved sensitivity for sepsis detection. This study demonstrates improved accuracy of early sepsis detection, based upon probability statistics, when MDW is combined with the earliest measured components of SIRS and qSOFA, which are often used to detect and assess the risk of sepsis during the initial ED encounter [13–15].

When extrapolated to a range of pre-test probabilities previously reported in ED patient populations [3–5, 22, 28, 29, 32, 33], MDW enhances the diagnostic performance of various SIRS combinations (Fig. 2). Just as importantly, a normal MDW value helps to identify those less likely to be septic, and for whom alternative diagnostic testing and treatments may be appropriate. When coupled with any of the other clinical parameters that are available during the initial ED encounter, a normal MDW significantly reduces the odds of sepsis, and an abnormal MDW significantly increases the odds of sepsis. For instance, a common clinical presentation in the ED is a combination of abnormal WBC and tachycardia, which can occur in an array of acute illnesses, including non-infectious diseases (trauma, cardiovascular disease) as well as sepsis [34]. In patients with abnormal WBC and tachycardia (Fig. 2a), the probability of sepsis increases approximately 2.5-fold with abnormal MDW compared to normal MDW based on an ED population with sepsis prevalence of 8%. The effect is even greater among those presenting with abnormal WBC and tachypnea (Fig. 2b), a common presentation in the ED, wherein an abnormal MDW versus normal MDW confers an approximate 5-fold higher risk of sepsis.

Importantly, approximately 31% of septic ED patients presented with a normal WBC at the time of admission. These patients represent a diagnostic dilemma because they are often deemed by healthcare providers to be at low risk of sepsis. However, a normal WBC with elevated MDW, compared to normal MDW alone, increases the probability of sepsis approximately 7-fold. Another perspective being that three in every four patients (i.e., 89/120) who presented initially with normal WBC and were diagnosed with sepsis within 12 h of ED admission could have been identified during the initial encounter based on elevated baseline MDW.

Similar to SIRS, qSOFA has poor sensitivity and specificity for early sepsis detection [19]. As was demonstrated for each SIRS criterion, an addition of MDW improves the diagnostic accuracy of qSOFA criteria for early sepsis detection in the ED. Of note, altered mental status and hypotension, the parameters differentiating qSOFA from SIRS, were uncommon in our ED sepsis population (Table 3). MDW was shown to synergize with altered mentation for sepsis detection (Fig. 3a), and when MDW was in the normal range, the probability of sepsis was lower in those with hypotension (Fig. 3b). The inability of qSOFA to distinguish infected from non-infected ED patients and the low prevalence of qSOFA criteria among septic patients explains why qSOFA alone is ineffective for guiding early sepsis treatments in the ED [17, 19, 20]. We have previously reported that MDW elevation is highly predictive of infection, which explains the observed improvement in sepsis detection (4-fold) when MDW is incorporated with qSOFA criteria during the initial ED encounter (Table 4).

How does MDW enhance early sepsis detection? The clinical conundrum encountered with sepsis during the initial ED encounter is the inability to distinguish infected from non-infected patients purely based on SIRS criteria. Changes in monocyte volume are observed in response to pro-inflammatory signals from infectious organisms, referred to as pathogen-associated molecular patterns (PAMPs) [35]. Thus, MDW may reflect a change in circulating monocyte volume corresponding with a transition from localized to systemic inflammatory response to sepsis due to interaction with circulating PAMPs. This could explain why MDW is more specific for infection, and given that monocytes are capable of amplifying immune responses, the activation of monocytes corresponding with elevated MDW could portent subsequent organ failures. Indeed, elevated MDW at the time of ED admission was highly predictive (71% sensitivity) of progression from infection to Sepsis-3 (with organ failures) within 72 h of ED admission [21].

There are several limitations to the study. We did not extend the analysis of sepsis progression beyond 12 h of ED admission. The inclusion of such patients would have reduced false negatives based upon 12-h sepsis analyses, which would have further improved the apparent performance of MDW when combined with SIRS and qSOFA parameters. The analyses provided herein are based on certain assumptions, such as sepsis prevalence in the ED ranging from 0 to 18%, and adult ED patient characteristics commonly encountered in large metropolitan hospitals located in the Midwestern and Northeastern USA from which the data was derived. Two of three academic centers were tertiary care providers (Ohio State University, Hackensack Medical Center), whereas one center (University of Pittsburg Medical Center, Shadyside) represents community hospital. Although the study was representative of seasonal changes in sepsis (e.g., pneumonia is more common in winter), sepsis etiologies and risk likely vary on a regional basis, as indicated by observed regional variations in sepsis mortality within the USA [36]. Additional prospective clinical studies will be required to validate the performance of MDW for early sepsis detection in other ED populations, including pediatric populations and in regions with different sepsis characteristics.

## Conclusions

MDW augments diagnostic accuracy for early sepsis detection when used in conjunction with clinical parameters that are widely available during the initial ED patient encounter during which sepsis screening and early detection are a high priority. Based on our analysis of data obtained from three large academic centers, the inclusion of MDW during the initial evaluation of ED patients enhances the odds of early sepsis detection by 6-fold for Sepsis-2 and 4-fold for Sepsis-3. We propose a clinical role of MDW to supplement current clinical parameters used to screen for sepsis, in essence, serving as a fifth SIRS criteria or a fourth qSOFA criteria, to enhance the early detection of sepsis in the ED.

## Data Availability

De-identified data from the ED patient population is not publicly available. However, elements of the datasets analyzed during the current study are available from the corresponding author on reasonable request.
